# Cell Surface Shaving-Based Proteomic Profiling of the Surfaceome in Pathogenic Microorganisms

**DOI:** 10.3390/ijms27021048

**Published:** 2026-01-21

**Authors:** Dorota Satala, Katarzyna Kowalik, Justyna Karkowska-Kuleta

**Affiliations:** 1 Department of Comparative Biochemistry and Bioanalytics, Faculty of Biochemistry, Biophysics and Biotechnology, Jagiellonian University in Krakow, Gronostajowa 7, 30-387 Krakow, Poland; 2 Doctoral School of Exact and Natural Sciences, Faculty of Biochemistry, Biophysics and Biotechnology, Jagiellonian University in Krakow, Gronostajowa 7, 30-387 Krakow, Poland

**Keywords:** cell surface shaving, surfaceome, pathogenic microorganisms, bacteria, host–pathogen interactions

## Abstract

The microbial cell wall is a dynamic structure responsible for maintaining the shape and integrity of the cell. It is involved in the processes of cell growth, reproduction and division, protection against environmental stress factors, bidirectional selective transport of various molecules, and interactions with other microorganisms inhabiting a particular ecological niche. The cell surface of microorganisms pathogenic to humans is also responsible for constant and direct contact with the host during the initiation and further development of the infection, and proteins exposed on the surface play a key role in this phenomenon. The set of proteins exposed on the cell surface is collectively referred to as the surfaceome. In surfaceome studies, the cell surface shaving technique is increasingly used while maintaining cell integrity, which, in addition to providing insight into the composition of surface proteins, also makes it possible to track the interactions of pathogen molecules with host molecules. This experimental approach will be described in this review with examples of the most relevant groups of identified microbial proteins involved in the host–pathogen interactions.

## 1. Introduction

Microorganisms demonstrate significant molecular diversity, reflected in the wide array of proteins they produce, which enable them to interact dynamically with their surrounding environment, detect external stimuli, and respond adaptively to variable conditions. This molecular repertoire includes surface structures, signaling components, metabolic enzymes, chaperones, and effector proteins that collectively support environmental sensing, communication, and survival in complex and adverse ecological niches [1]. The cell surface of microorganisms inhabiting the host organism, including both commensal and pathogenic species, serves as the primary structural interface mediating direct and constant interactions not only with the host, but also with other cohabiting microorganisms. Importantly, this outermost layer of microbial cells plays a critical role during infection, acting as the first place of contact with host cells, tissues, and immune defense molecules [2,3]. It is involved in a wide range of biological processes, including adhesion to host cells, evasion of immune responses, and initiation of signaling pathways that influence both microbial behavior and host physiology. In pathogenic microorganisms, the cell surface is often equipped with specialized molecules such as adhesins, invasins, and surface-associated enzymes or receptors that facilitate colonization and contribute to virulence [4,5]. Among these are moonlighting proteins, which have emerged as a notable focus of current research. These proteins are defined by their ability to carry out two or more unrelated biological functions despite being encoded by a single polypeptide chain. Such functional diversity often reflects activity in distinct cellular compartments or physiological contexts and does not arise from gene fusion, alternative splicing, or proteolytic processing [6]. The versatility of moonlighting proteins underscores their significance in cellular regulation and highlights the growing interest in elucidating the molecular mechanisms that enable their multifunctionality and involvement in the interactions between pathogenic microorganisms and host [4,6,7]. Consequently, the characterization of detailed composition and functional dynamics of microbial cell surfaces is crucial for understanding host–pathogen interactions and the mechanisms underlying infectious disease. To characterize the proteome of microbial cell surfaces (defined also as surfaceome), the surface shaving technique combined with shotgun proteomic approach has been extensively employed for protein identification. This method involves targeted proteolysis, in which specific proteases (e.g., trypsin, proteinase K, and others) selectively cleave surface-exposed regions of proteins embedded within the microbial surface barrier (cell envelope/cell wall/surface coat), making possible their subsequent identification with tandem mass spectrometry. Although shaving of bacterial and fungal cells can be hindered by the intricate, multilayered architecture and biochemical composition of their cell walls, such as the dense peptidoglycan network in Gram-positive bacteria or the polysaccharide-rich matrix in fungi, which can limit protease accessibility and reduce the efficiency of released protein fragments, these structural constraints also enhance the utility of the technique for selectively detecting proteins that are genuinely surface-exposed and play a key role in interactions with molecules in the surrounding environment. Therefore, the aim of this review is to provide a comprehensive overview of the identification of surface-exposed proteins in microorganisms pathogenic for humans, with a focus on information obtained with the use of cell surface shaving technique. The discussion encompasses selected bacterial, fungal, and protozoan species of clinical relevance and particular emphasis is placed on the functional potential of the identified proteins, including their candidacy as vaccine-included antigens and their roles in host–pathogen or microbe–microbe interactions. Given their extracellular localization and involvement in processes associated with virulence, these proteins represent promising targets for therapeutic intervention and immunological prevention of infectious diseases.

Beyond prior descriptive reviews, this synthesis provides a conceptual framework that shifts the focus from “what is present on the surface” to “how surface-exposed molecules collectively operate and remodel under biological pressure”. Specifically, we highlight (i) the collective and partially redundant action of recurrent moonlighting proteins, (ii) surfaceome remodeling as a dynamic, condition- and morphotype-dependent process, and (iii) interkingdom and host–pathogen interfaces in which mixed communities and adsorption of host factors reshape apparent surface exposure, together with contemporary validation and false-positive control logic that improves biological interpretability [8,9].

## 2. Cell Surface Shaving in Identification of Surface Exposed Proteins in Bacteria

Bacterial pathogens span a broad range of envelope architectures, and these structural differences are a primary determinant of which proteins are accessible to proteolytic shaving. In Gram-positive species, the absence of an outer membrane and the relatively direct exposure of the peptidoglycan-associated proteome generally favor efficient recovery of cell-wall-anchored and non-covalently associated proteins. By contrast, Gram-negative envelopes introduce an additional permeability barrier and compartmentalization (outer membrane/periplasm), which can bias datasets toward exposed outer-membrane constituents and require careful balancing of protease conditions against cell integrity [10,11].

**Figure 1:**
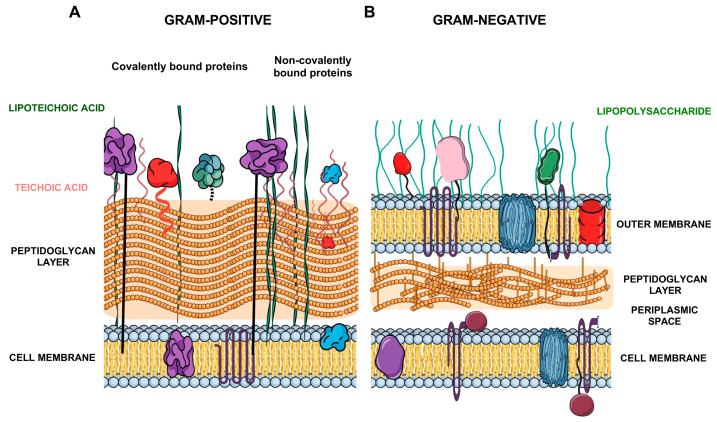
Schematic representation of the cell wall architecture in (**A**) Gram-positive and (**B**) Gram-negative bacteria. The diagram illustrates the multilayered organization of the bacterial cell envelope, highlighting the thick peptidoglycan (PG) layer in Gram-positive bacteria and the dual-membrane structure with a thin peptidoglycan layer in Gram-negative bacteria. Protein localization is indicated, including peptidoglycan-associated surface proteins, membrane-anchored proteins, and proteins exposed on the outer surface. This schematic emphasizes structural constraints relevant to surface shaving and the accessibility of surface-exposed proteins for proteolytic analysis. The illustration was created in the Mind the Graph Platform, available at www.mindthegraph.com.

The bacterial cell wall and the complexity of its architecture play a crucial role in shaping the composition of surface proteins accessible to proteolysis (Figure 1A,B).

Due to the presence of a thick peptidoglycan (PG) layer, Gram-positive bacteria possess a structurally robust cell wall that contributes significantly to their integrity and resilience. This characteristic has made them a crucial point in numerous studies investigating cell surface protein composition and model organisms for surface proteomics research [12,13]. Within this cell wall structure surface-exposed bacterial proteins can be covalently and non-covalently bound (Figure 1A). Many proteins are covalently anchored to the PG through LPXTG motifs (a conserved amino acid motif in Gram-positive bacterial surface proteins that is recognized and cleaved by sortase enzymes to anchor proteins to the cell wall), including adhesins and invasins involved in the interactions with the host. Other proteins are associated non-covalently through specific binding domains to peptidoglycan or teichoic acids, including moonlighting proteins of cytoplasmic origin [14,15]. Due to the lack of outer membrane structure, proteases can directly access proteins exposed on or embedded in the PG layer, making surface shaving particularly effective [10]. Cell walls of Gram-negative bacteria present much more complex challenge due to their double-membrane organization. Their cell wall consists of the cytoplasmic membrane, a thin peptidoglycan layer in the periplasmic space, and an outer membrane, enriched with lipopolysaccharide (LPS) (Figure 1B), thereby reducing the accessibility of proteins to applied proteolytic enzymes [11]. Therefore, the surface shaving process is mainly focused on β-barrel outer membrane proteins (OMPs), anchored lipoproteins, and secreted proteins [13]. Thus, the surface localization and structural exposition of protease-sensitive regions make porins such as OmpA, OmpC, and OprF highly detectable in proteolytic shaving experiments [11,13].

One of the earliest applications of shaving-based proteomics has been the comparative analysis of bacterial surfaceomes across clinical isolates and mutant strains, providing insights into how genetic variability translates into differences in adhesion, immune evasion, and virulence potential. In *Staphylococcus aureus*, surface-shaving combined with quantitative proteomics identified over 1200 proteins, with ~20 consistently differing relative to the reference strain Newman [13]. Differentially abundant proteins included clumping factors (ClfA, ClfB), protein A (SpA), the extracellular adherence protein (Eap/Map), and immune evasion factors such as SdrE, while moonlighting proteins such as elongation factor Tu (EF-Tu) were also detected [13]. In a recent, diagnostic-oriented multi-strain study of *S. aureus* clinical isolates, shaving data were further integrated with pangenome-based filtering and k-mer analysis to prioritize conserved, species-specific, and structurally accessible peptide targets [16]. In that multi-strain dataset (11 clinical strains), LC–MS/MS (high performance liquid chromatography-coupled tandem mass spectrometry) approach identified 873 proteins in total; after replicate- and strain-level filtering, 388 proteins were retained as common across all strains. Subsequent pangenome- and k-mer–based prioritization yielded 26 surface-exposed quasi-prime heptamer peptides mapped to 15 proteins, including known virulence-associated candidates (e.g., PbpA, Sbi, Asp23) and additional targets such as GAPDH and previously uncharacterized proteins encoded by SAUSA300_1904 and SAUSA300_1685 [16]. Ythier et al. combined surface shaving with transcriptomic profiling to characterize LPXTG-anchored proteins in *S. aureus* [17]. Identified proteins included clumping factors (ClfA, ClfB), fibronectin-binding proteins (FnbA, FnbB), serine-aspartate repeat proteins (SdrC, SdrD, SdrE), and iron-regulated surface determinants (IsdA, IsdB). Expression of these adhesins was shown to be tightly controlled by the agr quorum-sensing system and iron availability, highlighting the regulatory plasticity of the *S. aureus* surfaceome [17].

An important methodological milestone was provided by Olaya-Abril et al. [18], who optimized the shaving protocol for *Streptococcus pneumoniae*, a species prone to autolysis during proteolytic digestion. By adjusting growth conditions and digestion times, the authors minimized cytoplasmic contamination and maximized recovery of bona fide surface proteins. In total, 49 proteins were identified in the reference strain R6 and 114 in four clinical isolates, including well-known virulence factors such as CbpA (Spr1995), PhtD (Spr0907), PhtE (Spr0908), protease PrtA (Spr0561), adhesins PsaA (Spr0112) and PcpA (Spr1945), and the essential cell-wall hydrolase PcsB (Spr2021). Several ABC transporters (e.g., Spr1707, Spr1382, Spr1918) were also detected and confirmed by immunoreactivity assays. This study not only validated shaving as a robust approach for pneumococcal antigen discovery but also expanded the catalog of proteins with potential diagnostic or vaccine relevance [18].

Extending these analyses to mycobacteria, surface-shaving proteomics has revealed striking remodeling events in biofilm adaptation. In *Mycobacterium marinum* surface-shaving proteomics revealed distinct biofilm-specific remodeling: compared to planktonic cells, submerged and pellicle biofilms showed enrichment of oxidative stress-response enzymes (e.g., KatG, SodA, aAhpC, AhpD), mycolic acid biosynthetic enzymes (e.g., FadD32, KasA, KasB, and InhA), as well as PE/PPE adhesins (e.g., PPE31, PE5) and ESX secretion components. In addition, numerous cytoplasmic proteins with potential moonlighting functions (e.g., EF-Tu, GroEL1/GroEL2), were consistently detected on the surface of biofilm cells. By contrast, planktonic cells were relatively enriched in proteins linked to central metabolism and translation [19].

Extending this approach to Gram-negative bacteria has further demonstrated its versatility. In *Capnocytophaga canimorsus*, a commensal of dog oral flora and an emerging human pathogen, proteomic analysis identified 75 surface-exposed proteins, the majority encoded by polysaccharide utilization loci (PULs). These included multiple glycosidases and carbohydrate transporters predicted to target host-derived glycans, highlighting the central role of PUL-encoded proteins in shaping the Gram-negative surfaceome and revealing a distinctive adaptation of this species to carbohydrate-rich environments [20].

### Surface Shaving as a Technique for Identifying Bacterial Antigens for Vaccine Development

The most impactful application of shaving-based proteomics lies in the identification of antigenic proteins and the discovery of vaccine candidates. Unlike other uses of the method, this approach directly targets proteins exposed on the bacterial surface and thus accessible to the host immune system, providing an experimental shortcut to rational vaccine design.

Rodríguez-Ortega et al. [21] pioneered this strategy in the Gram-positive pathogen *Streptococcus suis*, identifying 46 surface-exposed proteins using shaving-based proteomics. These proteins largely fell into adhesion-related factors (including LPXTG-anchored substrates), nutrient acquisition systems (e.g., ABC transporters), and lipoproteins and membrane proteins. A follow-up study confirmed the value of this approach for antigen discovery and highlighted proteins such as Sao and Sat among the surfaceome of *S. suis*, reinforcing the potential of shaving proteomics for the identification of novel vaccine candidates [22]. Later, the strategy was extended to human clinical isolates [23]. In one study, 131 predicted surface proteins were identified across six serotype 2 strains, including known protective antigens such as Sao, Sat, SsnA, HP0197, SsPepO, and suilysin (Sly), while other major virulence factors like muramidase-released protein (Mrp) and extracellular protein factor (Epf) were not detected [23]. A complementary exoproteome analysis confirmed the consistent detection of cytoplasmic moonlighting proteins (e.g., enolase (Eno), glyceraldehyde 3-phosphate dehydrogenase (GAPDH), and fructose-1,6-bisphosphate aldolase (Fba)), which were recognized by convalescent human sera, supporting their relevance during natural infection [24].

A pan-surfomic analysis of 16 clinical isolates of *Streptococcus pneumoniae* identified 254 surface-associated proteins, including many well-established virulence factors and protective antigens [25]. These included classical vaccine-relevant proteins (e.g., PspA, CbpA/PspC, PhtD, PsaA), metal transporters (e.g., PiaA and PiuA), and the cell division protein PcsB. Importantly, analysis of their distribution across clinical isolates revealed a conserved core set of proteins: both PspA and CbpA/PspC were present in 15 of 16 isolates, as were the metalloproteinase ZmpB, IgA1 protease, the β-galactosidase precursor, the phage-encoded adhesin PblB, and PcsB. The broad representation of these proteins across diverse isolates underscores their central role in pneumococcal pathogenesis and highlights them as promising targets for vaccine development [25]. The protective value of PspA and PspC has been further confirmed in preclinical studies: immunization of humans with recombinant PspA elicited antibodies that protected mice against lethal pneumococcal challenge [26], while vaccine formulations combining multiple pneumococcal proteins, including PspA, demonstrated robust protection in murine models [27,28]. These findings underscore that shaving-based antigen discovery can be directly linked to protective outcomes in animal models.

Application of shaving to *Streptococcus agalactiae* (group B *Streptococcus*, GBS) uncovered a panel of surface-exposed proteins, including known adhesins and transporters, as well as a previously uncharacterized protein, SAN_1485, belonging to the serine-rich repeat family [14]. This protein family is typically involved in adhesion to host cells and biofilm formation, suggesting a role in colonization and persistence. The detection of SAN_1485 by shaving highlighted its genuine surface exposure and pointed to its potential as a novel vaccine candidate against GBS [14].

In the case of *Enterococcus faecalis*, surface-shaving proteomics combined with immunoreactivity screening led to the identification of 23 immunogenic proteins [29]. These comprised a broad repertoire of surface-associated factors, including enzymes involved in cell wall remodeling such as peptidoglycan-binding protein LysM, D-alanyl-D-alanine carboxypeptidase, and low-affinity penicillin-binding protein 5, folding catalysts such as PpiC-type peptidyl-prolyl cis-trans isomerase, and metabolic or housekeeping proteins like the 50S ribosomal protein L2. In addition, secreted and membrane-associated factors including an SCP-like extracellular protein, NLPA lipoprotein, and ABC-type transporters were also identified [29].

Beyond streptococci and enterococci, additional study has highlighted the potential of shaving in mycobacteria. Recent work has extended protease shaving to *Mycobacterium tuberculosis*, where quantitative mass spectrometry identified multiple surface-exposed PE/PPE proteins, including PPE18, PPE32, PPE38, PPE60, PE12 and PE23. Functional analyses confirmed surface accessibility of PPE18, a component of the M72/AS01E vaccine candidate [30].

Further broadening the taxonomic spectrum, additional Gram-positive pathogens have been interrogated. Studies on less-characterized Gram-positive species have further broadened the scope of surfaceome-based vaccinology. In *Trueperella pyogenes*, surface-shaving proteomics identified 140 surface-associated proteins across 15 clinical isolates, including cell wall-anchored proteins with atypical motifs (LAXTG and LSXTG), several conserved lipoproteins, and secreted proteins. Among them, the cholesterol-dependent cytolysin pyolysin (PLO) was detected in all isolates and ranked as a top vaccine candidate, together with additional conserved surface proteins predicted to be highly antigenic [31].

A recent refinement of the methodology was introduced by Sadones et al., who combined shaving with subtractive immunoproteomics (SUPRA) in *S. aureus* [32]. This workflow enabled the detection of more than 70 surface-associated proteins, among which several were prioritized based on reactivity with opsonic sera. By integrating SUPRA, the authors were able to minimize false-positive identifications typically associated with surface shaving and focus on antigens with genuine surface localization and immunological relevance. A central finding was the identification of AdcAau, a zinc-binding lipoprotein belonging to the AdcABC transporter system, which plays a key role in metal ion homeostasis [32]. Functional assays demonstrated that antibodies raised against AdcAau mediated efficient opsonophagocytic killing of *S. aureus*. Importantly, these antibodies also cross-reacted with homologous proteins in *Enterococcus faecium* (AdcAfm) and *E. faecalis*, thereby exhibiting cross-opsonic activity across multiple Gram-positive pathogens [32].

Although most work has focused on Gram-positive species, extension of shaving to Gram-negative bacteria has yielded equally significant findings. In *Burkholderia cenocepacia*, shaving combined with epitope prediction and immunological validation revealed 16 experimentally confirmed surface proteins; among them, OmpA-like proteins BCAL2958 and BCAL2645 and the PspA-like BCAL2022 were strongly reactive with sera from cystic fibrosis patients, underscoring their clinical relevance as vaccine antigens [33]. Similarly, in *Bordetella pertussis*, shaving-based proteomics identified 126 surface-associated proteins, including major outer membrane virulence factors such as Pertactin (Prn), Filamentous hemagglutinin (FhaB), Tracheal colonization factor (TcfA), Vag8, and BrkA, together with toxins like pertussis toxin subunit PtxA and adenylate cyclase toxin CyaA. In addition, several conserved housekeeping proteins with moonlighting functions, including elongation factor Tu (EF-Tu) and GroEL, were consistently detected, highlighting their potential as host-interacting antigens [34].

Collectively, these studies establish shaving as a cornerstone of surfaceome-based vaccinology. By profiling proteins directly on intact bacterial cells, the method enables rational selection of adhesins, transporters and multifunctional enzymes as vaccine candidates. Several proteins first discovered through shaving, such as PspA and PorA, have progressed into experimental vaccines or licensed formulations, providing direct evidence of clinical translation [26,28,35]. Others, including PspC in pneumococci and Ace/EfbA in enterococci, remain at the preclinical stage but continue to demonstrate strong protective potential.

## 3. Cell Surface Shaving in Identification of Surface Exposed Proteins in Fungi

Fungal pathogens possess thick, highly glycosylated and dynamically remodeled cell walls, making surface accessibility strongly dependent on growth conditions, morphology, and environmental stress. As a result, shaving profiles often reflect not only protein abundance but also epitope masking/unmasking within the polysaccharide matrix and variability in how deeply proteases can penetrate the wall. Consequently, fungal shaving datasets are best interpreted as “accessibility maps” of the wall surface under defined conditions rather than exhaustive inventories of all wall-associated proteins [36,37].

**Figure 2:**
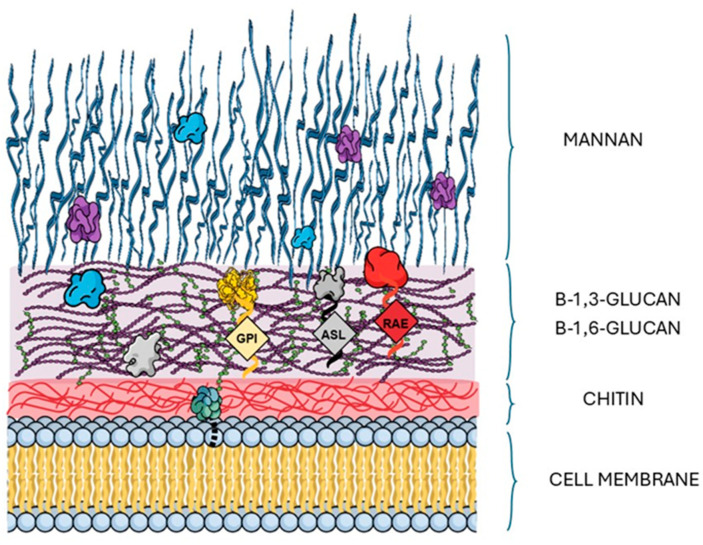
Schematic representation of the cell wall architecture of *Candida albicans*. The diagram illustrates the multilayered organization of the fungal cell wall and the localization of associated proteins, including those embedded within the glucan–chitin matrix, covalently linked via glycosylphosphatidylinositol (GPI) anchors, proteins connected through alkali-sensitive linkages (ASL), reducing agent-extractable (RAE) proteins, as well as non-covalently associated proteins exposed on the cell surface. This scheme highlights structural features relevant to surface shaving and the accessibility of proteins for proteolytic analysis. The illustration was created in the Mind the Graph Platform, available at www.mindthegraph.com.

The cell wall of *Candida albicans* exhibits a complex architecture composed of chitin, β-1,3- and β-1,6-glucans, mannans, and proteins (Figure 2). Within the polysaccharide matrix, which forms the backbone of the cell wall, the innermost layer is composed of chitin cross-linked with β-1,3-glucan and β-1,6-glucan, whereas the outer layer is enriched with mannans. Embedded within this network is a diverse set of proteins that regulate adhesion, enzymatic modification, and overall cell wall integrity [8]. Cell wall proteins may be covalently attached to the polysaccharide matrix through diverse types of anchors, the diversity of which can affect their extraction. The most common are glycosylphosphatidylinositol (GPI)-anchored proteins, secreted with a signal sequence and covalently linked to β-1,6-glucan [37]. This group includes major adhesins such as Als3, which mediates strong adhesion and invasion, as well as Hwp1, a hypha-specific adhesin capable of cross-linking to host cell proteins, and Eap1, which also contributes to adhesion. Another class comprises proteins with internal repeats (Pir proteins), covalently attached to β-1,3-glucan through an alkali-labile bond. Although best characterized in *Saccharomyces cerevisiae*, Pir proteins have also been identified in *Candida*. Their functional diversity has been linked not only to cell wall integrity but also to morphogenesis and antifungal resistance. Despite their covalent attachment, Pir proteins can be extracted using mild alkali treatment [33]. In addition to alkali-soluble cell wall proteins (ASPs), reducing agent-extractable proteins (REPs) can be isolated using β-mercaptoethanol treatment [34]. A third group consists of proteins integrated into the polysaccharide matrix without covalent linkage. This group includes also moonlighting proteins, which exert different functions depending on their localization or the environmental conditions [37].

The surfaceome of fungal pathogens has been extensively explored using shaving-based proteomics, which provides direct evidence of proteins exposed on the cell wall under conditions preserving cellular integrity. This approach has been particularly valuable for the characterization of fungal species, where dynamic remodeling of the cell surface is intricately linked to morphological transitions and adaptation to host niches.

One of the first large-scale studies applied trypsin shaving to yeast, hyphal and biofilm forms of *C. albicans*, leading to the identification of approximately 130 proteins [38]. These included known adhesins (e.g., Als1, Als3), cell wall remodeling enzymes (e.g., Cht3, Bgl2, Mp65), and moonlighting proteins (e.g., Eno1, GAPDH (Tdh3), Hsp70). Biofilm cells were enriched in adhesins (Als1, Als3) and remodeling enzymes (Mp65), whereas yeast cells displayed markers of commensal growth including Ywp1. These results demonstrate that the surfaceome composition varies markedly with morphology and growth conditions, reflecting fungal adaptability.

Follow-up studies expanded this repertoire substantially, identifying more than 900 proteins across yeast and hyphal forms of *C. albicans* [39]. Hyphal cells were enriched in virulence-associated factors (e.g., Hyr1, Sod5, Sap10, Als3 and Rbt1), while yeast cells prominently displayed commensal-associated markers (e.g., Ywp1 and Pir1). Stress-related proteins (including members of the Hsp70 family) and metabolic enzymes like Eno1 and Tdh3, were reproducibly detected in both morphologies. Their consistent presence supports their classification as moonlighting proteins with adhesive or immune-modulatory functions. Analyses of surfaceomes from mutant strains lacking selected wall proteins (*pst3*Δ/Δ, *tos1*Δ/Δ, *orf19.3060*Δ/Δ, *orf19.5352*Δ/Δ) further revealed additional molecules such as Ali1 and Mci4, showing that altered wall architecture can unmask proteins relevant to fungal adaptation [39].

An integrative study combining shaving-based proteomics with secretome analyses identified more than 570 proteins [37]. Among them were cell wall constituents such as Ecm33, Pga4 and the glucan-remodeling enzyme Phr2. Previously described moonlighting proteins were again detected. Comparative evaluation of the surfaceome and secretome revealed a substantial overlap, suggesting that many proteins are simultaneously wall-associated and secreted. Stress conditions further promoted the exposure of proteins such as Ecm33, Phr2, Hsp70, Eno1 and Tdh3. Their increased abundance under oxidative, osmotic and pH stress highlighted the plasticity of the fungal surfaceome in supporting persistence in hostile environments [37].

While large-scale inventories highlight the diversity and plasticity of the fungal surface, more recent studies have turned to mechanistic questions of how proteins are anchored and externalized. Karkowska-Kuleta et al. demonstrated that the major adhesin Als3 anchors Eno1 to the fungal surface, enabling interactions with host ligands including plasminogen and kininogen [40]. These interactions influence host proteolytic cascades and illustrate how moonlighting proteins acquire adhesive or immune-modulatory functions once externalized. Shaving thus provides both comprehensive protein inventories and crucial evidence of extracellular exposure, forming a basis for functional studies.

Surface shaving has likewise been applied to non-*albicans Candida* species. In yeast-like forms of *C. parapsilosis*, abundant proteins included a chitinase-like molecule, glyceraldehyde-3-phosphate dehydrogenase (Tdh3), and an inducible acid phosphatase, whereas *C. tropicalis* yeast cells exposed a constitutive acid phosphatase together with pyruvate decarboxylase (Pdc11) and Tdh3. Pseudohyphal forms of *C. parapsilosis* were enriched in the mannoprotein Mp65, a chitinase and the GPI-anchored transglycosylase Crh11, while pseudohyphae of *C. tropicalis* displayed Rbt1, a hyphally induced wall protein and members of the Als adhesin family. In addition to these proteins, several other covalently attached and atypical wall proteins were detected, further illustrating the remodeling capacity of the wall proteome under filament-inducing conditions [41]. Growth in RPMI medium increased the proportion of GPI-anchored proteins to ~50% in *C. parapsilosis* and ~30% in *C. tropicalis*, reflecting remodeling of the wall in filament-inducing conditions [41].

A comparative study of *Candida glabrata* (recently reclassified as *Nakaseomyces glabratus*), *C. parapsilosis* and *C. tropicalis* under infection-mimicking conditions (artificial saliva, AS; artificial urine, AU; vagina-simulative medium, VS; and anaerobic YPD, AN) identified 53, 37 and 13 surface proteins, respectively [42]. The highest numbers were consistently found under anaerobic growth, whereas the lowest were observed in VS for *C. glabrata* and *C. parapsilosis* and in AU for *C. tropicalis*. Differential exposure of moonlighting proteins was evident across media: in *C. glabrata*, several enzymes appeared only in AS, VS, AU or AN compared to the reference medium; in *C. parapsilosis*, no moonlighting proteins were detected after growth in VS; and in *C. tropicalis*, only three moonlighting proteins (Pdc11, Eno1, Tdh3) were reproducibly found, with increased surface abundance of Eno1 in AS and Tdh3 in VS. Collectively, these data demonstrate that surface exposure of moonlighting proteins is strongly niche-dependent, with anaerobic growth consistently yielding the richest surface proteomes [42].

Shaving has also been used to confirm the presence of individual adhesins. In *C. glabrata*, the epithelial adhesin Epa6 was detected at the cell surface after contact with fibronectin, complementing earlier biochemical evidence of its role in host binding [43]. Similarly, in *C. parapsilosis*, two proteins—CPAR2_404800 and CPAR2_404780—were identified and subsequently shown to function as adhesins interacting with epithelial and endothelial cells as well as extracellular matrix ligands, including fibronectin and vitronectin [44]. In both cases, shaving provided critical confirmation of protein exposure at the fungal surface, supporting later functional characterization.

Surface shaving has also been extended to filamentous fungi. In *Aspergillus fumigatus*, resting conidia were analyzed by combining classical HF–pyridine extraction with trypsin-based shaving, resulting in the identification of 148 surface-associated proteins, of which 48 were uniquely detected by the shaving approach [45]. Among these, RodA was confirmed as the dominant hydrophobin of the rodlet layer, while CcpA emerged as a previously uncharacterized protein abundant at the conidial surface. Deletion of CcpA altered the surface proteome profile, exposing additional proteins normally shielded in wild-type cells. This change correlated with enhanced activation of neutrophils and dendritic cells, reduced epithelial damage, and a marked loss of virulence in immunosuppressed mice. Furthermore, CcpA proved to be immunogenic, eliciting memory T cell responses in healthy human donors. Together, these observations established CcpA as a novel determinant of immune evasion and virulence in *A. fumigatus* and illustrated how shaving can uncover critical pathogenicity factors in mold conidia [45].

## 4. Cell Surface Shaving of Fungal Extracellular Vesicles

In addition to the cellular surface itself, increasing attention is being directed toward extracellular vesicles (EVs), that are structures of nanometer size released by all cell types. These structures, typically ranging from 30 to 1000 nanometers in diameter, are enclosed by a lipid bilayer and serve as transporters for the selective export of bioactive molecules, including proteins, lipids, and nucleic acids. Most likely the molecular cargo of EVs is not passively acquired but rather suggests regulated sorting mechanism that corresponds to the physiological or pathological state of the originating cell [46,47,48]. Thus, EVs represent a dynamic and functionally diverse component of the extracellular proteome, with emerging roles in intercellular communication, immune modulation, and biomarker discovery [49]. The localization of specific proteins on the surface of EVs plays a critical role in determining their functional properties, including their potential application as biomarkers and as targeted delivery vehicles for molecular cargo. Accordingly, comprehensive EV proteomic analyses should incorporate spatial profiling of protein topology, distinguishing between surface-exposed and luminal components as included in the recommendations of the International Society for Extracellular Vesicles [50]. This distinction is essential for the understanding of the mechanisms of vesicle biogenesis, intercellular communication, and selective molecular trafficking. In the case of EVs released by microorganisms, there are still very few studies that specifically target surface molecules of EVs and their identification. Typically, the procedure for identifying vesicular proteins is based on complete degradation of their structure and identification of all vesicular proteins. For some microorganisms, such as the fungus *Cryptococcus neoformans*, hydrolysis of EV surface proteins using proteinase K was performed to subsequently demonstrate, using concanavalin A binding and flow cytometry, that EVs are decorated with mannoproteins, which, after proteinase degradation, are no longer displayed on the EV surface [51]. However, in the study by Rizzo et al. [51] those proteins after shaving procedure were not individually identified, but the whole proteome of fungal EVs was further identified with classical shotgun approach after disrupting the vesicle membrane and releasing transported proteins, which were then digested with trypsin.

Cell surface shaving of fungal EVs was also applied for the identification of proteins decorating those structures released by *C. parapsilosis*, *C. tropicalis* and *C. glabrata* [52]. In this study 22, 30 and 32 proteins from *C. parapsilosis*, *C. tropicalis* and *C. glabrata* EVs, respectively, were identified as vesicular surface-exposed proteins (Table 1). In addition, other proteins were identified in EVs of these three species by complementary approaches, with consideration given to their membrane localization or incorporation within EVs [52,53]; however, these proteins are not listed here, as the summary presented in Table 1 focuses exclusively on proteins identified using the shaving approach.

A subset of these proteins comprises typical membrane-associated or cell wall-anchored proteins, some of which participate in structural remodeling or maintenance of the cell envelope. In contrast, another subset consists of moonlighting proteins, which are frequently detected on the cell surface and may also be incorporated into EVs. Representative members of this latter group include, among others, enolase, alcohol dehydrogenase, and glyceraldehyde-3-phosphate dehydrogenase for *C. tropicalis* EVs, and for *C. glabrata* vesicles hexokinase, transaldolase, pyruvate decarboxylase, pyruvate kinase and 6-phosphogluconate dehydrogenase (Table 1; [52]). Among the typical proteins associated with the *Candida* EV surface are several cell wall structural and functional components, including exo-1,3-β-glucanase Xog1, cell wall enzyme 1,3-β-glucosyltransferase Bgl2, GPI-anchored cell wall protein Ecm33, cell wall protein with similarity to glucanases Scw4, cell surface mannoprotein Mp65 and many others (Table 1; [52]). The shared presence of key proteins like Scw4 and numerous glucanases (e.g., Bgl2, Xog1, Exg1) and glycosidases (e.g., Phr2) underscores a conserved, fundamental role for EVs in trafficking components necessary for maintaining and modifying the fungal cell wall. Trafficking these enzymes via EVs is an efficient strategy, allowing for the modification of the outermost cell layer. Furthermore, it provides a mechanism for cooperative remodeling within a fungal community, such as a biofilm, where EVs released from one cell can contribute to the cell wall maturation of other cells in the microenvironment. Furthermore, also fungal extracellular proteases have been identified in this location, including *C. tropicalis* secreted aspartyl protease Sapt4 and *C. glabrata* putative aspartic protease yapsin Yap3. Such diversity of proteins on the vesicle surface may indicate their multifunctionality, potentially related to non-classical pathways of moonlighting protein transport, translocation of entire vesicles through the cell wall, extracellular proteolytic activity, or adhesive properties and the ability to interact with host molecules or with other microorganisms. Consistently, EV-associated export is frequently discussed as one plausible conduit for signal-peptide-lacking moonlighting proteins, although additional non-classical translocation and surface-capture mechanisms are likely to coexist [8].

## 5. Cell Surface Shaving in Identification of Surface Exposed Proteins in Protozoa and Helminths

Protozoan parasites frequently display highly specialized and stage-specific surface architectures, including dense surface coats and rapid turnover or shedding of exposed molecules, which can strongly bias shaving outputs. Experimental design should therefore control life-cycle stage and parasite viability, and it should distinguish genuinely exposed parasite antigens from host-derived material that may adsorb to parasite surfaces or to infected host cells. These constraints make protozoan shaving particularly informative for host–parasite interfaces, but they also place a premium on stringent controls and careful biological interpretation [54,55].

In protozoan parasites, the application of cell surface shaving has proven effective for identifying surface-exposed proteins. A pioneering study on *Trypanosoma cruzi* epimastigotes resulting in the identification of more than one thousand proteins associated with the plasma membrane [56]. Among these were members of the trans-sialidase family, as well as conserved moonlighting proteins such as Eno and GAPDH. Additional stress-related and structural proteins, including Hsp70, Hsp90, and tubulin α/β, together with histones (H2A, H2B, H3, H4), were also reproducibly detected, underscoring the broad spectrum of molecules exposed at the parasite surface [56].

A further protozoan example comes from *Plasmodium falciparum*, where surface shaving was used to profile parasite-derived proteins accessible at the membrane of infected red blood cells (pRBCs) [54]. Using complementary shaving-based strategies combined with quantitative LC–MS/MS, the authors detected major variant surface antigen families involved in immune evasion and sequestration, including PfEMP1 (VAR2CSA), as well as members of the RIFIN, SURFIN and MC-2TM families. Importantly, the study also highlighted less variable, putative single-copy candidates recurrently detected across approaches, such as PIESP2 and PfJ23 (Hyp16), alongside exported proteins linked to Maurer’s clefts that participate in trafficking to the pRBC surface. Selected candidates were further validated by immunofluorescence microscopy and flow cytometry, with loss of signal after trypsin treatment supporting true surface accessibility. Together, these findings illustrate how shaving can support antigen discovery in protozoan infections by combining broad coverage of surface-exposed molecules with prioritization of more conserved targets [54].

A complementary example comes from *Schistosoma mansoni*, a helminth parasite whose tegument represents the major interface with the host bloodstream. Castro-Borges et al. applied enzymatic shaving on live adult worms using trypsin and phosphatidylinositol-specific phospholipase C (PiPLC) [55]. This approach revealed a set of parasite proteins exposed at the tegument surface, including Sm200, Sm25, Sm29, annexins and calpain, as well as GPI-anchored enzymes such as alkaline phosphatase and ADP-ribosyl cyclase. Remarkably, several host proteins, including CD44, CD48, CD90 and complement components C3 and C4, were also detected on the worm surface, indicating active acquisition of host molecules during intravascular migration. These findings not only provided insights into tegument biology but also suggested novel vaccine candidates such as Sm29, Sm200 and annexins.

Importantly, the protozoan and helminth literature is currently less extensive than the evidence base available for bacteria and fungi, in part because many parasites exhibit highly stage-specific surface architectures, dense surface coats/tegumental interfaces, and rapid turnover or shedding of surface molecules, all of which can bias protease accessibility and complicate interpretation. Consequently, shaving-based datasets in these organisms typically require particularly stringent viability controls and orthogonal validation to distinguish genuinely exposed parasite antigens from stress-released intracellular proteins or host-derived material adsorbed onto the surface. These studies illustrate that, similar to bacteria and fungi, surface shaving in protozoa provides valuable insights into the molecular landscape of parasite–host interfaces. By identifying proteins directly exposed to the extracellular milieu, this methodology contributes to our understanding of protozoan and helminth pathogenesis and may guide the identification of novel targets for diagnostics or therapeutic intervention.

## 6. Cell Surface Shaving in Studying Interactions

Moonlighting proteins constitute one of the most frequently reported groups among surface-exposed molecules identified by shaving. Their recurrent detection across independent studies suggests that their presence in the pathogenic cell wall is not accidental. Importantly, several of these proteins have also been detected during interactions with host cells, reinforcing the view that they represent genuine and functionally relevant components of the surfaceome. Mechanistically, the recurrent surface detection of classically cytosolic moonlighting proteins can be rationalized by unconventional export and retention processes. Because these proteins typically lack N-terminal signal peptides, they may reach the extracellular space via non-classical secretion routes, including release as cargo within extracellular vesicles, direct membrane translocation involving transport activities, or “lock-type” surface capture events associated with cell division. Additional, still-debated contributors include altered membrane lipid asymmetry (e.g., flippase-dependent effects) and post-translational modifications that may modulate trafficking or surface compatibility. Once externalized, moonlighting proteins can be retained through non-covalent binding to cell wall/envelope components or by physical trapping within dense polysaccharide matrices; importantly, secondary re-adsorption from the extracellular milieu after release from damaged cells has also been proposed as a complementary route. Together, these mechanisms provide a parsimonious framework for interpreting why moonlighting proteins repeatedly emerge in shaving datasets and why their apparent exposure can vary with growth state and stress conditions [8]. To illustrate this, Table 2 provides examples of fungal moonlighting proteins identified by shaving, together with their validated host binding partners and reported affinities, thereby complementing the proteomic surveys presented in this section.

Beyond mapping pathogen-derived proteins, shaving-based proteomics has also proven valuable for exploring the dynamic interface between microbes and their hosts, by revealing both changes in microbial surface composition and host proteins adsorbed onto fungal cells during infection. A representative example comes from *C. albicans* hyphae exposed to human serum [9]. In this setting, proteomic analyses identified 372 fungal proteins together with 214 human proteins bound to the cell wall. The fungal dataset included 147 proteins annotated as surface-associated and 52 described as immunogenic, among them 23 GPI-anchored molecules, the complement-evasion factors Pra1 and Gpd2, and seven plasminogen-binding enzymes (Adh1, Eno1, Fba1, Pgk1, Tdh3, Tef1, Tsa1) [60]. Importantly, twelve proteins—including Pra1, Sap5 and Tef1—were detected exclusively on hyphae grown in serum, underscoring the remodeling triggered by host factors. On the host side, nearly all major complement components (C3, C4, factor B, factor H) and coagulation factors (fibrinogen, plasminogen, prothrombin) were recovered, along with immunoglobulins, apolipoproteins, serpins, and cytoskeletal proteins [9]. The composition of this surfaceome varied with complement activity: cells incubated in normal serum (NS) displayed 372 surface proteins, compared to only 134 in heat-inactivated serum (HIS), with 43 unique to each condition. Deposition of complement proteins such as C3 was further validated by immunofluorescence [9].

A similar dual perspective was obtained when examining the impact of neutrophil extracellular traps (NETs) on *C. albicans* [59]. Comparative analyses of hyphae before and after neutrophil contact revealed profound remodeling of the fungal surface. Classical wall proteins such as Als3 and Cht2 were detected only after interaction with neutrophils, while moonlighting proteins (Eno1, triosephosphate isomerase 1 (Tpi1), phosphoglycerate mutase 1 (Gpm1), elongation factor 2) were consistently present but became more abundant or accessible. In parallel, shaving detected a wide range of NET-derived proteins deposited on the fungal surface in both yeast and hyphal forms [59]. These included myeloperoxidase (MPO), neutrophil elastase (HNE), proteinase-3, cathepsin G, lactoferrin, histones and the antimicrobial peptide LL-37, all stably associated with hyphae. Biochemical assays confirmed that binding was mediated not only by Als3 but also by moonlighting proteins such as Eno1, Gpm1 and Tpi1, which acted as fungal receptors with nanomolar affinities. Remarkably, adsorption of NET proteins was not fungicidal but instead enhanced virulence by promoting epithelial cell damage [59].

Shaving has also clarified host-induced changes in other fungi. In *C. glabrata*, fibronectin binding was shown to trigger selective surface exposure of the adhesin Epa6, detectable only in the presence of fibronectin [43]. This underscores the dual value of the method: detecting host proteins bound to the fungal surface and revealing pathogen proteins whose exposure depends on host contact.

In filamentous fungi, shaving has illuminated virulence-associated proteins of *A. fumigatus*. Trypsin-based analyses identified conidial surface proteins involved in adhesion and immune recognition, including the hydrophobin RodA, the mannoprotein CcpA, histone H2B, and moonlighting enzymes such as Eno1 and Tpi1 [45]. A comparative study with related nonpathogenic species (*A. fischeri*, *A. oerlinghausenensis*, *A. lentulus*) further revealed 62 proteins uniquely associated with *A. fumigatus*, of which 33 influenced macrophage survival or epithelial invasion [65]. Among these, the glucoasparaginase AspA emerged as a key virulence determinant, promoting persistence and pro-inflammatory cytokine responses. Collectively, these findings highlight how surfaceome profiling explains the higher pathogenic potential of *A. fumigatus* compared to its close relatives.

This plasticity of the fungal surfaceome has also been mirrored in bacteria. A surface-shaving proteomic approach combined with tandem mass tag (TMT) labeling was applied to *S. haemolyticus* in order to compare the bacterial surfaceome before and after colonization of human keratinocytes (HaCaT cells) [66]. The analysis revealed marked differences in the abundance and accessibility of surface-exposed proteins between these two conditions. Proteins associated with adhesion and biofilm formation, such as the autolysin Atl, fibronectin-binding protein FnbA and the biofilm-associated protein Bap, were enriched after contact with host cells, indicating that colonization triggers reorganization of the bacterial surface architecture. Similarly, nutrient acquisition factors, including the oligopeptide permease OppA and the manganese-binding protein MntC, were upregulated at the bacterial surface. In contrast, several proteins decreased in abundance, notably the metabolic enzyme Pgk, the cell division regulator EzrA, and surface-associated factors such as SsaA and MetQ, suggesting a strategic reallocation of surface components during host interaction [66]. Although this study focused on bacterial proteins rather than host molecules bound to the microbial surface, it clearly demonstrates that surface shaving can capture the dynamic plasticity of the bacterial surfaceome in response to host contact, a process likely to facilitate colonization and persistence within host tissues.

A related study on *S. pneumoniae* applied shaving combined with LC–MS/MS to compare bacterial surfaceomes under conditions simulating contact with epithelial cells and exposure to human plasma [67]. The results revealed adaptive remodeling of both the capsule and the protein surface composition depending on the growth environment. Eight experimental conditions were examined, including global host interaction assays, direct contact with epithelial cells or macrophages, combined cell interaction models, and growth in plasma-like medium. Across nearly all settings, proteins associated with capsule biosynthesis and cell wall remodeling dominated the surfaceome [67]. The capsule biosynthesis protein CapD and the zinc metalloproteinase ZmpB were repeatedly among the most abundant hits, together with the β-galactosidase precursor and a cell wall-associated serine proteinase precursor. Specifically, CapD was highly represented in global host interaction and epithelial contact experiments, whereas ZmpB prevailed in macrophage-associated conditions and plasma-like medium [67]. In addition, the β-galactosidase precursor was a frequent marker in mixed interaction assays, while the serine proteinase precursor was prominent in epithelial and plasma-mimicking environments. Collectively, these findings demonstrate that while the pneumococcal surfaceome varies in response to distinct host-derived signals, a conserved set of proteins—particularly CapD and ZmpB—consistently define the surface profile and likely contribute to pathogenesis across multiple host niches [67].

A further example comes from *S. aureus*, Dreisbach et al. [68] applied surface shaving to profile human serum proteins bound to the bacterial cell surface, providing one of the first global views of this type of interaction. More than 40 host proteins were identified, including complement components (C3, C4, C7, factor H and its related proteins), fibrinogen, vitronectin, plasminogen, α2-macroglobulin, and the platelet-derived antimicrobial peptide platelet factor 4 (PF4). Interestingly, the binding of PF4 and the acute-phase protein ITIH4 was observed only for the Newman strain, but not for USA300, highlighting strain-specific differences in the acquisition of host factors. These findings demonstrate that shaving-based proteomics can uncover not only the repertoire of host proteins coating the bacterial surface but also the variability of such interactions between clinical lineages of the same pathogen.

Although most studies have focused on Gram-positive bacteria, shaving has also been successfully applied to Gram-negative species, providing new perspectives on host exploitation and nutrient acquisition. An illustrative Gram-negative example comes from *C. canimorsus*, where surface shaving highlighted the functional role of PUL5 enzymes in host interaction. This locus was shown to mediate deglycosylation of host glycoproteins such as fetuin, enabling the bacterium to use host-derived glycans as a nutrient source. Mutants lacking PUL5 failed to grow on mammalian cells and displayed reduced survival in a mouse infection model, underscoring how surface-exposed carbohydrate-active enzymes directly contribute to host exploitation and virulence [20].

In addition to host–pathogen interactions, shaving-based proteomics has also been applied to study microbial consortia, where physical and biochemical interactions stabilize biofilms and enhance the survival of individual partners. This approach provides insights into the protein repertoire involved in fungal–bacterial cooperation. A prominent example is the dual-species biofilm formed by *C. albicans* and the periodontal pathogen *Porphyromonas gingivalis* [69]. Proteomic shaving revealed that several fungal adhesins and cell wall proteins, including Als3, Mp65 and Eno1, were strongly enriched at the fungal surface during mixed biofilm development [69]. These proteins interacted directly with bacterial virulence factors, notably gingipains (RgpA, RgpB, Kgp) that possess both proteolytic and hemagglutinin domains. Surface plasmon resonance assays confirmed the high affinity of Als3 and Mp65 for RgpA (KD ~ 10 nM), while Eno1 bound even more strongly (KD ~ 3 nM). Such interactions facilitated firm adhesion of *P. gingivalis* cells to fungal hyphae and created a protective niche for the anaerobic bacterium under oxic conditions. The fungal biofilm thus served not only as a structural scaffold but also as a microenvironment supporting the survival of an obligate anaerobe.

In a complementary study, shaving-based proteomics was combined with functional assays to investigate the role of the bacterial peptidylarginine deiminase (PPAD) in modulating fungal surface proteins [70]. A set of citrullinated *C. albicans* proteins was identified, including Als3, Mp65, Eno1, Eng1, Tdh3, Pga4 and heat shock proteins Ssa1/Ssa2. These post-translational modifications altered the interactions of *C. albicans* with human plasma proteins, reducing its ability to bind plasminogen and kininogen. Thus, the activity of a bacterial enzyme significantly reshaped the fungal surfaceome, indirectly influencing subsequent pathogen–host interactions.

Taken together, these studies establish cell surface shaving as a powerful strategy to unravel microbial–microbial interactions. Beyond identifying adhesive proteins that promote physical cross-linking between different species, this approach also uncovers fungal proteins subjected to enzymatic modification during interspecies contact. Such findings shed light on cooperative strategies among oral pathogens and emphasize the complexity of cross-kingdom biofilm formation.

## 7. Methodological Considerations, Advantages, and Limitations of Cell Surface Shaving

**Figure 3:**
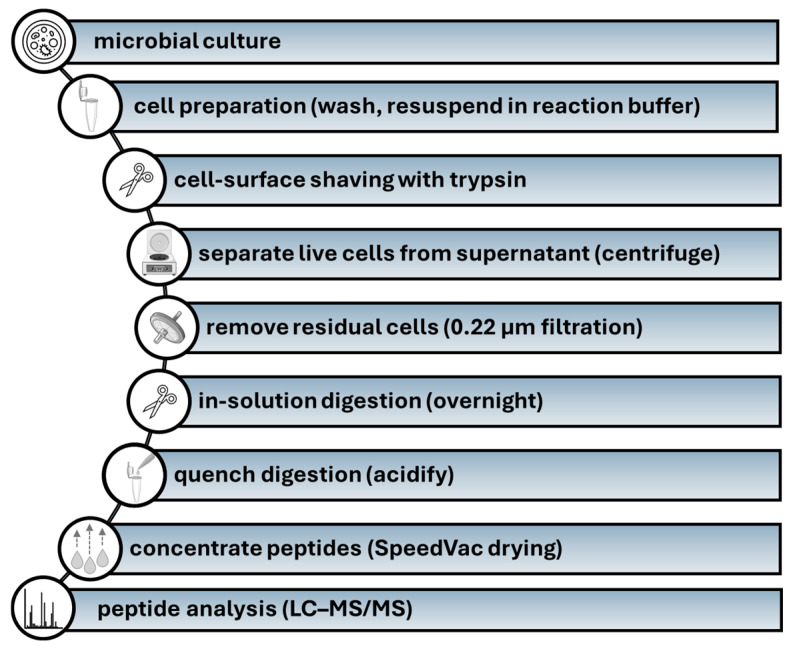
Stepwise schematic of the cell-surface shaving pipeline used for surfaceome discovery. LC–MS/MS-high performance liquid chromatography-coupled tandem mass spectrometry. The figure was partly generated using Servier Medical Art, provided by Servier, licensed under a Creative Commons Attribution 3.0 unported license.

To provide a framework for the methodological discussion below, Figure 3 outlines a typical cell-surface shaving workflow from culture and buffer exchange, through on-cell trypsinization and peptide cleanup, to LC–MS/MS analysis. At the same time, rigorous viability and contamination controls are essential, because even minor loss of membrane integrity can release abundant cytosolic proteins [71]: commonly used readouts include using impermeant dyes, complemented by colony-forming unit (CFU) counts [72,73]. Assay choice is typically tailored to the organism and the cultivation format, and culture-based readouts such as CFU are applied where appropriate [72,73]. In practice, membrane integrity is often assessed using dye combinations that discriminate live and compromised cells and samples showing evidence of permeabilization are excluded prior to downstream LC–MS/MS analysis [74]. Depending on the organism and growth form, these readouts are typically acquired by flow cytometry and/or fluorescence microscopy, providing a stringent pre-analytical filter for shaving datasets [74].

To validate genuine surface exposure, shaving datasets should be complemented with orthogonal assays performed on intact cells. (i) Immunofluorescence on non-permeabilized specimens (fluorescence microscopy or flow cytometry) can confirm extracellular epitope accessibility, provided that fixation/permeabilization is avoided and appropriate controls are included (secondary-only and isotype controls, and a cytosolic marker that should remain negative). (ii) Protease accessibility assays probe whether candidate proteins are digested from the outside: intact cells are briefly treated with an external protease (e.g., trypsin or proteinase K) and the target is assessed by immunodetection; a selective loss or size shift in the target signal, together with preserved cytosolic markers, supports surface exposure rather than cell damage. (iii) Protease protection assays extend this principle by comparing intact versus selectively permeabilized cells to distinguish truly external domains from proteins shielded within the wall/envelope or beneath protozoan surface coats. (iv) Western blot analysis of surface-enriched fractions (e.g., chemically surface-labeled/affinity-captured material or other surface-enrichment workflows) can further substantiate localization by demonstrating enrichment of the candidate alongside depletion of intracellular markers. Finally, integrating independent evidence streams—such as overlap between shaving and surface-labeling datasets—provides a practical criterion to increase confidence in genuine surface localization [75,76].

Interpretation must also account for method-intrinsic biases, including protease specificity (trypsin cleaves after lysine/arginine and may favor proteins enriched in these residues) and short incubation windows (typically 5–30 min) imposed to preserve cell integrity [10,77,78]. False positives can arise not only from subtle damage but also from bona fide surface-associated moonlighting proteins; therefore, no-protease controls that capture spontaneously released proteins are frequently used to flag candidates requiring cautious annotation [4,79,80,81]. Finally, the effective penetration depth of proteases is limited by surface-barrier architecture across microbes, which imposes an inherent accessibility bias in shaving experiments. In bacteria, fungi, and protozoa alike, dense wall/envelope or coat structures can sterically restrict enzyme access, leading to under-detection of deeply embedded, heavily glycosylated, or tightly associated proteins [8,36,37]. To place these trade-offs in context, a short comparative overview of cell surface shaving and commonly used surfaceome profiling strategies is provided in Table 3. This comparison highlights how differences in capture chemistry and accessibility translate into distinct strengths, biases, and recommended use cases.

As summarized in Table 3, complementary approaches such as biotinylation or other surface-labeling chemistries can extend coverage but may introduce steric effects or modify cleavage sites, and in some systems (e.g., *Candida*) avidin binding to the cell wall can confound interpretation [76,85]. Conversely, exoproteomics primarily captures secreted proteins, whereas shaving emphasizes proteins stably associated with the cell surface [82].

## 8. Conclusions and Future Perspectives

Cell surface shaving has become a widely applied strategy for characterizing microbial surfaceomes, revealing both conventional adhesins and unconventional moonlighting proteins, and providing functional insights into host–pathogen interactions. Across bacteria, fungi, and protozoa, shaving-based proteomics has not only cataloged surface-exposed proteins but also uncovered their dynamic remodeling under host and environmental pressures. These findings have firmly established shaving as a powerful experimental platform for dissecting microbial adaptation and virulence.

Future perspectives in surface-shaving proteomics point toward its growing role at the interface of basic research and clinical application. In vaccinology, combining shaving with advanced bioinformatics tools, such as epitope prediction algorithms and structural modeling, enables the prioritization of epitopes most likely to elicit protective immune responses [33]. Quantitative proteomic platforms such as TMT or iTRAQ further expand this potential, allowing comparative analyses across multiple strains and growth conditions to identify conserved antigens suitable for broad-spectrum vaccine development [13,66]. These strategies are expected to accelerate the design of multicomponent vaccines in which adhesins, transporters and moonlighting enzymes can be rationally combined to overcome redundancy in bacterial virulence factors [23,30,31]. An additional promising avenue is the use of shaving-based workflows to uncover cross-protective antigens with activity against multiple pathogens. The recent identification of AdcAau in *S. aureus* as an immunogenic antigen cross-reactive with enterococcal homologues exemplifies how shaving can inform pan-pathogen vaccine strategies, particularly against multidrug-resistant ESKAPE organisms [32].

Looking ahead, coupling surface shaving with emerging high-resolution proteomic modalities could further refine how microbial exposure is quantified and interpreted. Integration with single-cell proteomics may help resolve population heterogeneity, capturing rare but clinically relevant subpopulations and transient surface states that are averaged out in bulk measurements. Spatial proteomics applied to infected tissues and structured microbial communities could add anatomical and microenvironmental context, linking surface-exposed antigens to specific host niches and local immune pressures. Finally, epitope prioritization could increasingly leverage machine-learning models that integrate shaving-derived quantitative evidence with sequence- and structure-based features, supporting more systematic selection of candidates for downstream validation. In addition, the integration of multi-omics approaches for the investigation of intercellular interactions, especially those that account for the extracellular vesicle surfaceome, should be further developed to enhance our understanding of cell-to-cell communication [49,94].

Beyond vaccinology, shaving-based proteomics also holds promise for clinical diagnostics and therapeutic monitoring. Because the surfaceome represents the pathogen–host interface, dynamic changes in its composition may provide accessible signatures for rapid detection, species differentiation and even tracking of antimicrobial resistance. In line with these findings, recent studies suggest that advanced proteomic analyses may facilitate the identification of putative biomarkers, as exemplified by *S. pneumoniae*, where several highly ranked proteins, many of which are surface-associated and exhibit virulence-related or antigenic properties, have been proposed as species-specific markers, although their broader applicability requires further validation [83]. Longitudinal analyses of clinical isolates could reveal how pathogens remodel their surfaceomes during infection and treatment, offering insights into adaptive strategies under drug pressure and during infection development, reflecting the dynamics of the interactions between host and pathogen, and such approaches may ultimately contribute to personalized infection management, including the development of vaccines tailored to specific populations or circulating strains [49,95,96,97,98,99]. Finally, the extension of shaving-based proteomic approaches to host tissues and polymicrobial communities represents a promising avenue for future research. By selectively profiling surface-exposed proteins, this strategy may enable the simultaneous characterization of pathogen antigens, host-accessible microbial factors, and interface-associated host proteins. In complex polymicrobial settings, shaving-based analyses could further contribute to elucidating cooperative and competitive interactions among coexisting microorganisms, as well as their collective influence on host responses. Such interactions are increasingly recognized as critical determinants of infection progression and outcome; nevertheless, the inherent complexity of these systems underscores the need for careful methodological optimization and rigorous validation to ensure accurate interpretation of surfaceome data and biological relevance [100,101,102].

Together, these perspectives underscore the translational value of shaving-based proteomics. By bridging fundamental studies of microbial surfaceomes with vaccine discovery, diagnostics and therapeutic applications, this methodology is poised to become a keystone of both pathogen biology and infectious disease management.

## Figures and Tables

**Table 1:** Mass spectrometry identification of abundant proteins in EVs from three different *Candida* species after vesicle surface shaving with trypsin with their functional categorization (based on the data and detailed protein list from [52]).

Functional Category	*C. parapsilosis*	*C. tropicalis*	*C. glabrata*
Cell wall integrity & remodeling	Pga45, Xog1, Scw4, Ecm33, Phr2, Bgl2, Mp65, Mnn7, Pir1, Ywp1	Phr1, Mp65, Atc1, Bgl2, Phr2, Scw4	Cwp1, Scw4, Exg1/Spr1, Exg1, Ath1, GAS-1 homolog, Ecm33
Adhesion	putative adhesin-like, Op4	Rbt1, Op4, putative adhesin-like, Hyr3, Pga17	
Carbon metabolism & energy production		Adh1, Adh2, Eno1, Gca1, Tdh3, Pgk1, Pma1, Cdc19	Acs1, Hxt7, Hxk2, Tal1, Tdh3, Cys3, Pck1, Eno1, Adh1, Fba1, Pgk1, Pdc1, Cdc19, Gnd1
Protein synthesis & translation			P0, L1, Eft2, Tif1, Hsc82, Asc1
Proteolysis & protein modification	Prc3, Kex2	Cyp1, Sapt4	Yap3
Lipid metabolism		Plb3	
Phosphatases	Pho100	Pho113, Pho100	
Uncharacterized/Other	CPAR2_107330, CPAR2_108490, CPAR2_300570, Abg1, CPAR2_407280, CPAR2_807950, CPAR2_500500	CTRG_04138, CTRG_05676, CTRG_01989, CTRG_04834, CTRG_02903, CTRG_03859	CAGL0A02255g, Spg2, Pst2, Ygp1

**Table 2:** Fungal moonlighting proteins identified at the cell surface by shaving-based proteomics, with experimentally confirmed host binding partners and reported dissociation constants (*K*_D_). HK—high-molecular-mass kininogen, FXII—factor XII, PPK—prekallikrein, MPO—myeloperoxidase, HNE—elastase, LF—lactoferrin, LL-37—cathelicidin LL-37, HPG—plasminogen, VTR—vitronectin, FN—fibronectin, LAM—laminin, COL—collagen, ELA—elastin.

Moonlighting Protein	Confirmed Host Binding Partner(s)		*K*_D_ (M)	Reference
	*Candida albicans*			
enolase (Eno1)	plasma factors	HK	2.25 × 10^–7^	[57]
		FXII	7.33 × 10^–7^	[57]
		PPK	2.13 × 10^–7^	[57]
		HPG	1.20 × 10^−7^	[58]
	components of NETs	MPO	7.87 × 10^–8^	[59]
		HNE	3.61 × 10^–8^	[59]
		LF	7.22 × 10^–8^	[59]
	antibacterial peptide	LL-37	1.46 × 10^–8^	[59]
	extracellular matrix components	VTR	3.11 × 10^−8^	[58]
		FN	3.68 × 10^−8^	[58]
triosephosphate isomerase 1 (Tpi1)	plasma factors	HK	7.87 × 10^−8^	[57]
		FXII	7.21 × 10^–8^	[57]
		PPK	6.69 × 10^–7^	[57]
	components of NETs	MPO	4.50 × 10^–8^	[59]
		LF	1.63 × 10^–7^	[59]
	antibacterial peptide	LL-37	6.83 × 10^–8^	[59]
	extracellular matrix components	VTR	8.60 × 10^−8^	[60]
		FN	3.26 × 10^−7^	[60]
		LAM	7.39 × 10^−7^	[60]
		COL	1.09 × 10^−7^	[60]
		ELA	8.21 × 10^−7^	[60]
phosphoglycerate mutase 1 (Gpm1)	plasma factors	HK	4.79 × 10^–7^	[57]
		FXII	5.48 × 10^–7^	[57]
		PPK	3.97 × 10^–7^	[57]
	components of NETs	MPO	3.53 × 10^–7^	[59]
		LF	2.14 × 10^–8^	[59]
	antibacterial peptide	LL-37	5.98 × 10^–7^	[59]
	extracellular matrix components	VTR	2.73 × 10^–8^	[61]
		FN	2.02 × 10^–8^	[61]
glyceraldehyde 3-phosphate dehydrogenase (GAPDH)	plasma factors	HPG	1.50 × 10^−7^	[62]
	extracellular matrix components	VTR	4.42 × 10^−8^	[62]
glucose-6-phosphate isomerase (Gpi1)	plasma factors	PPK	6.09 × 10^–7^	[57]
*Candida parapsilosis*				
6-phosphogluconate dehydrogenase 1 (Gnd1)	plasma factors	HK	7.22 × 10^–9^	[63]
heat shock protein Ssa2	plasma factors	HK	6.93 × 10^–8^	[63]
		HPG	4.03 × 10^–7^	[63]
*Candida tropicalis*				
enolase (Eno1)	plasma factors	HK	1.42 × 10^–7^	[64]
		HPG	2.53 × 10^−7^	[58]
	extracellular matrix components	VTR	6.83 × 10^−7^	[58]
		FN	5.18 × 10^−8^	[58]
phosphoglycerate mutase 1 (Gpm1)	plasma factors	HK	5.81 × 10^–7^	[64]
*Candida glabrata*				
triosephosphate isomerase 1 (Tpi1)	extracellular matrix components	VTR	1.68 × 10^−7^	[60]
		FN	7.30 × 10^−7^	[60]
		LAM	2.53 × 10^−7^	[60]
		COL	2.14 × 10^−7^	[60]
		ELA	2.01 × 10^−8^	[60]
glyceraldehyde 3-phosphate dehydrogenase (GAPDH)	plasma factors	HPG	8.69 × 10^−9^	[62]
	extracellular matrix components	VTR	4.80 × 10^−8^	[62]

**Table 3:** Comparative overview of cell surface shaving and complementary surfaceome profiling approaches. The table summarizes each method’s core principle, major strengths, typical limitations/artifacts, and recommended use cases for identifying and validating genuinely surface-exposed microbial proteins. LC–MS/MS—high performance liquid chromatography-coupled tandem mass spectrometry, IF—immunofluorescence, WB—Western blot.

Principle	Key Advantages	Typical Limitations/Artifacts	Practical Use/Example	Reference
cell surface shaving				
brief incubation of intact/live cells with a protease → identification of protease-accessible peptides by LC–MS/MS	enriches for truly accessible regions; provides domain-level exposure information; strong for condition/morphotype comparisons (remodeling)	sensitive to micro-lysis (cytosolic carryover); accessibility/penetration bias depends on the surface barrier; protease-specific cleavage bias	prioritize vaccine candidates by focusing on peptides proven accessible on intact cells	[10,16,71,76,82,83]
surface biotinylation (NHS-biotin labeling)				
chemical labeling of accessible surface amines → affinity capture (pull-down) → LC–MS/MS	strong surface enrichment; no proteolysis required; scalable	steric hindrance possible; depends on accessible amines; membrane damage can cause intracellular labeling; *Candida*: avidin/streptavidin-related binding can confound	use overlap (biotinylation—shaving) to increase confidence in true surface localization	[16,76,84,85,86,87,88]
periodate–hydrazide (surface glycan capture)				
oxidize accessible glycans with periodate → couple hydrazide/biotin → enrich surface glycoprotein	targets glycoprotein-rich outer layers; useful when heavy glycosylation masks protease access	oxidation can alter structures; biased toward glycosylated proteins; misses non-glycosylated targets	map surface glycoproteins and compare epitope masking/unmasking across growth states	[76,89,90]
cell wall/envelope fractionation + proteomics				
mechanical/chemical fractionation of wall/envelope → LC–MS/MS of fraction proteins	often higher coverage of wall/envelope-associated proteins	cytosolic contamination if lysis occurs; does not directly prove surface exposure	build a comprehensive wall proteome, then validate exposure by shaving or labeling	[71,76]
exoproteomics/secretome (supernatant proteomics)				
LC–MS/MS of proteins found in the culture supernatant	excellent for secreted factors (enzymes, toxins); simple sampling	mixes secretion + shedding + lysis; does not directly report surface exposure	compare secretome vs. shaving to separate released proteins from stably surface-associated ones	[82,91,92]
surface-exposure validation assays				
independent assays that confirm extracellular epitope accessibility or enrichment in surface fractions (IF/protease accessibility/protease protection/surface-fraction WB)	increases confidence; distinguishes exposure vs. artifacts; supports mechanistic claims	requires reagents (e.g., antibodies); lower throughput than MS	validate a candidate by loss of signal after external protease treatment + WB enrichment in surface fraction	[9,54,93]

## Data Availability

No new data were created or analyzed in this study. Data sharing is not applicable to this article.

## Abbreviations

The following abbreviations are used in this manuscript:

COL	Collagen
ELA	Elastin
FN	Fibronectin
FXII	factor XII
GPI	Glycosylphosphatidylinositol
HK	High-molecular-mass kininogen
HNE	Elastase
HPG	Plasminogen
LAM	Laminin
LC–MS/MS	High-performance liquid chromatography–coupled tandem mass spectrometry
LF	Lactoferrin
LL-37	Cathelicidin LL-37
MPO	Myeloperoxidase
PG	Peptidoglycan
PPK	Prekallikrein
VTR	Vitronectin
